# Screening for type 2 diabetes: do screen-detected cases fare better?

**DOI:** 10.1007/s00125-017-4402-4

**Published:** 2017-08-23

**Authors:** Adina L. Feldman, Simon J. Griffin, Eva Fhärm, Margareta Norberg, Patrik Wennberg, Lars Weinehall, Olov Rolandsson

**Affiliations:** 1 MRC Epidemiology Unit, Institute of Metabolic Science, University of Cambridge, Cambridge, UK; 2 The Primary Care Unit, Institute of Public Health, University of Cambridge, Cambridge, UK; 3 Department of Public Health and Clinical Medicine, Family Medicine, Umeå University, 901 87 Umeå, Sweden; 4 Department of Public Health and Clinical Medicine, Epidemiology and Global Health, Umeå University, Umeå, Sweden

**Keywords:** Diabetes mellitus, Early diagnosis, Epidemiology, Mass screening, Public health

## Abstract

**Aims/hypothesis:**

We aimed to investigate whether diabetes cases detected through screening have better health outcomes than clinically detected cases in a population-based cohort of adults who were eligible to be screened for diabetes at 10 year intervals.

**Methods:**

The Västerbotten Intervention Programme is a community- and individual-based public health programme in Västerbotten County, Sweden. Residents are invited to clinical examinations that include screening for diabetes by OGTTs at age 30, 40, 50 and 60 years (individuals eligible for screening, *n* = 142,037). Between 1992 and 2013, we identified 1024 screen-detected cases and 8642 clinically detected cases of diabetes using registry data. Clinically detected individuals were either prior screening participants (*n* = 4506) or people who did not participate in screening (non-participants, *n* = 4136). Study individuals with diabetes were followed from date of detection until end of follow-up, emigration, death or incident cardiovascular disease (CVD), renal disease or retinopathy event, and compared using Cox proportional hazard regression adjusted for calendar time, age at detection, year of detection, sex and socioeconomic status.

**Results:**

The average age at diabetes diagnosis was 4.6 years lower for screen-detected individuals compared with clinically detected individuals. Overall, those who were clinically detected had worse health outcomes than those who were screen-detected (HR for all-cause mortality 2.07 [95% CI 1.63, 2.62]). Compared with screen-detected study individuals, all-cause mortality was higher for clinically detected individuals who were screening non-participants (HR 2.31 [95% CI 1.82, 2.94]) than for those clinically detected who were prior screening participants (HR 1.70 [95% CI 1.32, 2.18]). Estimates followed a similar pattern for CVD, renal disease and retinopathy.

**Conclusions/interpretation:**

Individuals with screen-detected diabetes were diagnosed earlier and appeared to fare better than those who were clinically detected with regard to all-cause mortality, CVD, renal disease and retinopathy. How much of these associations can be explained by earlier treatment because of screening rather than healthy user bias, lead time bias and length time bias warrants further investigation.

**Electronic supplementary material:**

The online version of this article (doi:10.1007/s00125-017-4402-4) contains peer-reviewed but unedited supplementary material, which is available to authorised users.

## Introduction

More than 1 million adults in the UK and 160,000 adults in Sweden are estimated to be living with undiagnosed diabetes [1], which is potentially detectable by screening. In screening for type 2 diabetes, one cluster-randomised controlled trial in a high-risk UK population (Anglo–Danish–Dutch study of intensive treatment in people with screen-detected diabetes in primary care [ADDITION]-Cambridge [2]) and a controlled trial in a high-risk Danish population (ADDITION-Denmark [3]) found no effect on mortality in the population after approximately 10 years. One cohort study in an average-risk UK population (the Ely cohort) reported a reduction of mortality in 1990–1999, but no effect 10 years later [4]. In the Ely cohort, the average lead time for a diabetes diagnosis following screening was estimated at 3.3 years, but this was not associated with lower incidence of adverse health outcomes for individuals detected earlier through screening [5]. A study in Sweden compared people with diabetes detected through an opportunistic screening programme with those detected clinically in the same eligible population and found no difference in age at diagnosis or any effect on health outcomes for screen-detected individuals [6]. However, in ADDITION-Denmark, a lead time of 2.2 years was associated with lower mortality and cardiovascular disease (CVD) risk among those in the screened group [7].

One review found that the positive predictive values of a single biochemical screening test for diabetes ranged between 24% and 48% [8], meaning that more than half of those with positive screening tests probably have only transient non-diabetic hyperglycaemia. Although it is known that, compared with normoglycaemia, those with non-diabetic hyperglycaemia have an increased risk of CVD and death [9], the fate of those with unconfirmed diabetes following a positive test result has not specifically been studied.

Following reports in the 1970s of relatively high mortality from CVD in the Swedish county of Västerbotten, a community public health intervention programme was launched [10]. The Västerbotten Intervention Programme (VIP) was first implemented in 1985 and reached full coverage in 1992. There is some evidence that the overall public health programme has had a positive impact on all-cause and CVD mortality [11]. VIP has both a community and an individual focus, with invitations to standardised health examinations in primary healthcare [10]. Crucially, these include OGTTs, which allows us to study the VIP as a model for an organised systematic universal diabetes population screening programme.

We aimed to investigate the association between screen detection of type 2 diabetes and all-cause mortality, CVD events, renal disease and retinopathy in this population-based cohort of adults eligible to be screened at 10 year intervals. The secondary aim was to investigate the rate of these outcomes in unconfirmed screen-positive cases.

## Methods

### The VIP

Since 1985, residents of Västerbotten County have been eligible for invitation to standardised health examinations at the age of 30 (until 1995), 40, 50 and 60 years, as previously described in detail [10]. Briefly, at every VIP examination, participants are asked to complete a comprehensive questionnaire that covers, among other things, lifestyle behaviour and health status; this, together with OGTT results, is used as the basis for a motivational health promotion dialogue. Individuals found to have non-diabetic hyperglycaemia receive referrals for a follow-up visit with a nurse, and individuals found to have diabetes are referred to primary care for confirmatory testing and treatment [10]. The overall rate of participation at the first eligible VIP examination over the study period has ranged from 48% to 67% [12]. Objectively measured data, such as BMI and blood glucose measurements, and associated questionnaire data collected in the VIP health examinations may be linked to local and national registers using the Swedish personal identification number [13].

Ethical approval for this study was granted by the Regional Ethical Review Board, Umeå, Sweden (Dnr 2013-395-31M, Dnr 2014-410-32M).

### Eligible study population

**Fig. 1:**
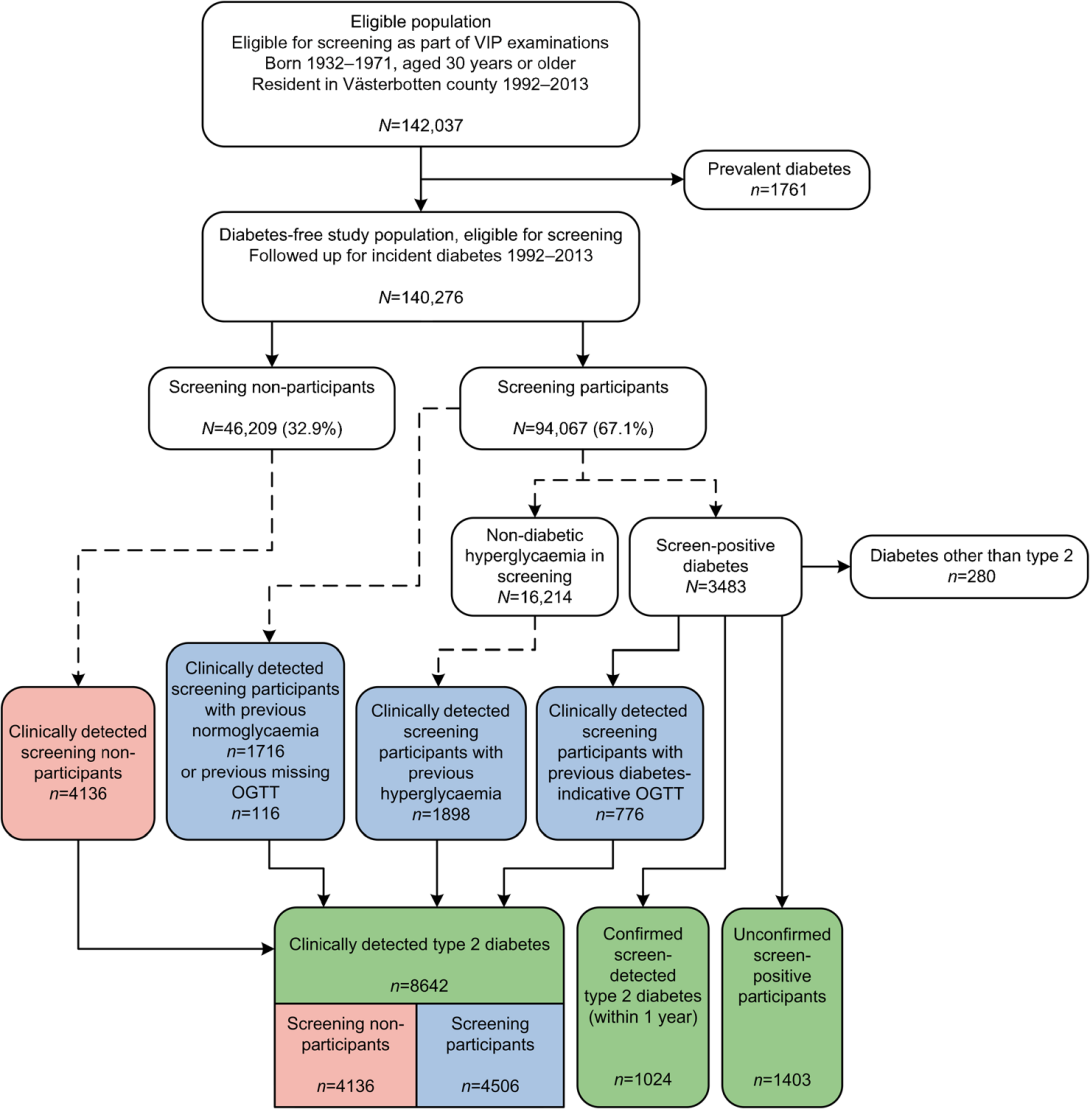
Flow chart describing the study population. The prevalent diabetes-free study population (*N* = 140,276) was eligible for screening; 46,209 individuals did not participate in screening and 94,067 did. Among the screening participants, 16,214 had non-diabetic hyperglycaemia (*n* = 1898 were later detected clinically) and 3483 screened positive for diabetes. Of those who screened positive, the diagnosis was confirmed within 1 year in 1024 individuals, and remained unconfirmed in 1403 (*n* = 280 were found to have diabetes other than type 2). A total of 776 individuals were clinically detected after having screened positive for diabetes more than 1 year previously, 1716 were clinically detected after having normoglycaemia in screening, and 116 were clinically detected after participating in a health examination without completing an OGTT. In total, among all screening participants, *n* = 4506 cases of diabetes were clinically detected, whilst in screening non-participants, 4136 cases of diabetes were clinically detected. Dashed lines indicate that downstream boxes do not include all individuals in upstream box, solid lines indicate that all individuals in upstream box are accounted for in downstream boxes

Eligible individuals (*n* = 142,037) were identified in the Population Register that is maintained by the Swedish Tax Agency [14]. They were resident in Västerbotten County between 1992 and 2013, born between 1932 and 1971, and aged 30 years or older with sufficient information available to enable record linkage to other population-based registers (Fig. 1). We excluded individuals who had a record of prevalent diabetes (*n* = 1761), leaving a study population of 140,276 individuals eligible for screening among whom incident diabetes was identified. We followed up the study individuals for detection of diabetes from 1 January 1992, their 30th birthday or the date of their move to Västerbotten County, until 31 December 2013, death or emigration, whichever came first.

### Screen-detected diabetes

In the study population, 94,067 individuals attended at least one VIP examination, which corresponds to a participation rate of 67.1% (Fig. 1). Each health examination included an OGTT using a 75 g oral glucose load [15]. In total, 16,214 individuals had a non-diabetic hyperglycaemic OGTT measurement (fasting or 2 h capillary plasma blood glucose of 6.1–6.9 mmol/l or 8.9–12.1 mmol/l, respectively), and 3483 individuals had an OGTT measurement indicative of diabetes (defined according to current diagnostic criteria as a fasting or 2 h capillary plasma blood glucose level of ≥ 7.0 mmol/l or ≥ 12.2 mmol/l, respectively) without any prior record of a diabetes diagnosis. Among participants with diabetes-indicative OGTT measurements, 1024 (29.4%) were confirmed as having type 2 diabetes within 1 year in at least one medical or prescription record (see details below); the majority were confirmed in either the Västerbotten County Medical Record system (*n* = 486, 47.5%) or the Diabetes Register in Northern Sweden (DiabNorth; *n* = 425, 41.5%); 1403 (40.3%) individuals did not have any record of a diabetes diagnosis besides one OGTT in the diabetic range and were, thus, classed as unconfirmed screen-positive participants (Fig. 1). In addition, 776 (22.3%) individuals had an OGTT indicative of diabetes but were only confirmed as having diabetes after more than 1 year and were thus considered to have clinically detected diabetes (see below), and 280 individuals had a medical record of a type of diabetes other than type 2, for example type 1 diabetes or gestational diabetes (Fig. 1).

### Clinically detected diabetes

In total, 8642 individuals with diabetes were identified in five sources of medical and prescription records (numbers and percentages refer to those with their earliest date of diagnosis in the source): (1) DiabNorth (*n* = 1205, 13.9%), a register of validated diabetes diagnoses in VIP until 2012 [16]; (2) the National Diabetes Register (*n* = 603, 7.0%), a resource linked to primary care that was initiated in 1996 [17] (coverage of the National Diabetes Register in Västerbotten County ranged between 50% and over 70% of individuals with diabetes registered [18]); (3) the Västerbotten County Medical Record system (*n* = 4984, 57.7%), which includes records of all primary care visits in the county since 2006, with partial coverage since 1993; (4) the Prescribed Drugs Register (*n* = 896, 10.4%), which includes records of all dispensed prescription drugs since 1 July 2005 (a diabetes record defined as dispensing of any drug with an Anatomical Therapeutic Chemical code A10) [19]; and (5) the National Patient Register, which includes all inpatient discharge records since 1987 (*n* = 858, 9.9%), with partial national coverage since 1964, and outpatient records since 2001 (*n* = 96, 1.1%) (diabetes defined as a record with a primary or contributory diagnosis of diabetes (ICD codes 250 [revision 9; www.icd9data.com/2007/Volume1] or E11, E13 or E14 [revision 10; www.who.int/classifications/icd/en/]) [20]. These sources were also used to confirm screen-detected diabetes cases (see above). Using capture–recapture [21], we estimate that 96.9% of diabetes cases in the population were identified using a combination of these sources.

Individuals with clinically detected diabetes were divided into two groups based on participation in VIP screening before the first detection of diabetes; VIP participants were further divided into three groups. A total of 4506 (52.1%) individuals with diabetes had participated in the VIP at least once before the date of detection of diabetes and had had either: (1) an OGTT measurement indicative of diabetes (if more than 1 year before detection, *n* = 776); (2) a non-diabetic hyperglycaemic OGTT (*n* = 1898); or (3) a normoglycaemic OGTT (*n* = 1716) (*n* = 116 had participated in the VIP but had missing OGTT data). The remaining 4136 (*n* = 47.9%) individuals with clinically detected diabetes never participated in VIP screening, despite being eligible to do so, before the detection of diabetes (Fig. 1).

### Events and outcomes

The primary outcome was date of death as identified by record linkage to the Total Population Register (Statistics Sweden) [14]. Secondary outcomes were dates of incident CVD events (myocardial infarction, heart failure, stroke or peripheral arterial disease), incident renal disease or incident retinopathy. All disease events were identified in the National Patient Register as well as the Cause of Death Register, which has had complete coverage since 1961 [22]. See electronic supplementary material (ESM) Table 1 for a detailed list of all the ICD-9 and ICD-10 codes used to classify events.

### Other variables

Sex and date of birth were taken from the Total Population Register [14]. Socioeconomic status (SES) was categorised into four levels (manual workers, non-manual workers, self-employed and undefined) in the 1990 census. For 1561 individuals who had missing information on SES, we imputed values using single imputation (the ‘ice’ command in Stata [version 14.2; StataCorp, College Station, TX, USA]) based on the variables sex, birth date, VIP participation (yes/no) and international migration status. Prior CVD events were ascertained in the National Patient Register and defined as above if they occurred before or on the same date as diabetes detection. For VIP participants, height, weight, BP and serum total cholesterol were objectively measured at VIP examinations [10]. BMI was calculated as the weight (kg) divided by height squared (m^2^). The VIP questionnaire includes the question, ‘how has your health been in the past year?’; we dichotomised the response alternatives ‘good’, ‘pretty good’, ‘somewhat good’, ‘pretty bad’ and ‘bad’ into ‘overall good’/‘overall bad’ [23].

### Statistical analysis

Individuals with diabetes were followed up from date of first detection of diabetes (i.e. the date of VIP screening for screen-detected diabetes, or the date of the first diabetes record for clinically detected diabetes) until 31 December 2013, date of death, emigration or date of outcome event depending on the model. Crude incidence rates and mortality rates were calculated as events divided by time at risk scaled to 1000 person-years. Directly standardised incidence rates and standardised mortality rates were calculated with weights derived from the follow-up time and age and sex distribution in the total study population (*n* = 140,276) as reference. Associations between mode of detection of diabetes and outcomes were assessed using Cox proportional hazard regression, generating HRs and 95% CIs. All models used calendar time as the underlying time scale and were adjusted for age at and calendar year of diabetes detection as continuous variables, sex and SES in 1990.

Differences in biomarkers measured at concurrent or previous VIP examination between individuals with screen-detected diabetes vs those with unconfirmed screen-positive and clinically detected diabetes who were screening participants were tested using the *t* test for continuous variables, and the *χ* ^2^ test for categorical variables. To disentangle the contributions of early treatment and length time bias to the difference in outcome rates between screen-detected and clinically detected individuals with diabetes who had been screening participants, we conducted two sensitivity analyses: (1) main analysis further adjusted for prior CVD event status; and (2) main analysis further adjusted for prior CVD event status, and the following variables measured at concurrent or previous VIP examination—BMI, diastolic and systolic BP, serum total cholesterol, self-reported health status and time from screening to diabetes detection in years. All analyses were performed in Stata version 14.2 (StataCorp).

## Results

We identified 9666 diabetes cases in total, constituting a cumulative incidence of 6.9% in the study population. Those with screen-detected diabetes were on average 4.6 years younger at diagnosis than those with clinically detected diabetes, 6.4 years younger than clinically detected individuals who were screening participants and 2.8 years younger than clinically detected individuals who were screening non-participants (Table 1). There was a substantial difference in the proportion of individuals who had experienced a CVD event prior to the date of detection of diabetes between those who had screen-detected diabetes (5.4%) and those with clinically detected diabetes (15. 2%). Among all individuals with clinically detected diabetes, 227 (2.6%) had a CVD event recorded on the same date as the date of detection of diabetes (data not shown).

**Table 1:** Descriptive statistics of participants with type 2 diabetes mellitus, VIP 1992–2013

Variable				Incident clinically detected diabetes		
	Total	Confirmed screen-detected diabetes	Unconfirmed screen-positive individuals	All	Screening participants	Screening non- participants
Total	11,069 (100.0)	1024 (9.3)	1403 (12.7)	8642 (78.1)	4506 (40.7)	4136 (37.4)
Men	6459 (58.4)	595 (58.1)	783 (55.8)	5081 (58.8)	2572 (57.1)	2509 (60.7)
Women	4610 (41.6)	429 (41.9)	620 (44.2)	3561 (41.2)	1934 (42.9)	1627 (39.3)
Age at detection, years						
Mean ± SD (median)	58.5 ± 9.7 (59.9)	55.1 ± 6.4 (59.8)	53.4 ± 8.0 (59.7)	59.7 ± 9.9 (60.2)	61.5 ± 9.0 (62.2)	57.9 ± 10.4 (58.4)
30–39	246 (2.2)	0 (0.0)	27 (1.9)	219 (2.5)	20 (0.4)	199 (4.8)
40–49	1410 (12.7)	82 (8.0)	220 (15.7)	1108 (12.8)	441 (9.8)	667 (16.1)
50–59	3296 (29.8)	335 (32.7)	406 (28.9)	2555 (29.6)	1222 (27.1)	1333 (32.2)
60–69	4494 (40.6)	607 (59.3)	750 (53.5)	3137 (36.3)	1851 (41.1)	1286 (31.1)
70+	1623 (14.7)	–	–	1623 (18.8)	972 (21.6)	651 (15.7)
Year of detection						
1992–1999	2012 (18.2)	217 (21.2)	369 (26.3)	1426 (16.5)	352 (7.8)	1074 (26.0)
2000–2006	3841 (34.7)	369 (36.0)	537 (38.3)	2935 (34.0)	1426 (31.6)	1509 (36.5)
2007–2013	5216 (47.1)	438 (42.8)	497 (35.4)	4281 (49.5)	2728 (60.5)	1553 (37.5)
SES in the 1990 census^a^						
Manual workers	5516 (49.8)	479 (46.8)	701 (50.0)	4337 (50.2)	2259 (50.1)	2089 (50.5)
Non-manual workers	4235 (38.3)	411 (40.1)	543 (38.7)	3285 (38.0)	1737 (38.5)	1534 (37.1)
Self-employed	744 (6.7)	82 (8.0)	103 (7.3)	562 (6.5)	305 (6.8)	264 (6.4)
Undefined	574 (5.2)	52 (5.1)	56 (4.0)	458 (5.3)	205 (4.5)	249 (6.0)
Prior CVD event^b^	1420 (12.8)	55 (5.4)	53 (3.8)	1312 (15.2)	673 (14.9)	639 (15.4)

Data are presented as *n* (%), unless otherwise stated

Unconfirmed screen-positive individuals only had a diabetic screening result, whereas confirmed screen-detected individuals had a diabetic screening result and a medical or prescription record of diabetes within 1 year. Those with clinically detected diabetes were identified in five sources of medical and prescription records, unrelated to screening

^a^SES imputed for *n* = 1561 individuals who had missing information

^b^Previous CVD events included those coinciding with the date of diabetes detection (*n* = 227)

Among clinically detected individuals with diabetes who were screening participants, those who had a previous diabetic or non-diabetic hyperglycaemic screening result were diagnosed on average 6.3 and 6.9 years, respectively, after their last screening. Those who had previously had a normoglycaemic screening result were diagnosed with diabetes on average 10.3 years after their last screening; among individuals who had screened negative for diabetes or non-diabetic hyperglycaemia, 13 (0.76%) were diagnosed with diabetes within 1 year (data not shown). Overall, among screening participants, those with screen-detected diabetes had similar mean levels of self-reported bad health and BP to those with clinically detected diabetes and had had a diabetic or non-diabetic hyperglycaemic OGTT result at previous screening (Table 2). For serum total cholesterol, the average levels were also very similar (≤ 0.2 mmol/l difference between confirmed screen-detected diabetes and the other groups), although the differences were statistically significant. Compared with screen-detected diabetes, mean BMI was 1.1 kg/m^2^ lower among clinically detected individuals who were screening participants. Among screen-detected individuals with diabetes, 443 (43.3%) reported a family history of diabetes in the VIP questionnaire; the corresponding number was 1519 (33.7%) among clinically detected individuals who were screening participants (data not shown).

**Table 2:** Characteristics measured at concurrent or previous screening among individuals with type 2 diabetes mellitus who were screening participants, VIP 1992–2013

Variable				Incident clinically detected diabetes, screening participants (*n* = 4561)							
	Confirmed screen-detected diabetes (*n* = 1024)	Unconfirmed screen-positive individuals (*n* = 1403)		All (*n* = 4506)		Previous diabetes-indicative OGTT >1 year before detection (*n* = 776)		Previous non-diabetic hyperglycaemia (*n* = 1898)		Previous normoglycaemia (*n* = 1716)	
			*p* value		*p v*alue		*p* value		*p* value		*p* value
Age at concurrent or previous screening (years)	55.1 (6.4)	53.4 (8.0)	–	53.4 (7.9)	–	54.2 (7.3)	–	54.3 (7.2)	–	51.9 (8.7)	–
Time from screening to first register detection (years)	0.1 (0.2)	–	–	8.1 (4.9)	–	6.2 (4.1)	–	6.9 (4.5)	–	10.3 (4.9)	–
BMI (kg/m^2^)	30.8 (5.6)	27.8 (4.8)	< 0.001	29.7 (4.7)	< 0.001	30.3 (5.0)	0.048	29.9 (4.7)	< 0.001	29.1 (4.4)	< 0.001
Self-reported overall bad health, *n* (%)	397 (38.8)	459 (32.7)	0.002	1795 (39.8)	0.741	323 (41.6)	0.455	757 (39.9)	0.588	662 (38.6)	0.993
Systolic blood pressure (mmHg)	140.9 (18.6)	136.6 (20.5)	< 0.001	139.2 (18.7)	0.010	140.8 (19.2)	0.967	140.3 (18.6)	0.394	137.0 (18.4)	< 0.001
Diastolic blood pressure (mmHg)	86.2 (10.6)	83.2 (11.5)	< 0.001	85.2 (11.1)	0.007	86.0 (10.8)	0.638	85.4 (10.6)	0.051	84.3 (11.3)	< 0.001
Serum total cholesterol (mmol/l)	5.6 (1.1)	5.5 (1.2)	0.011	5.8 (1.2)	< 0.001	5.8 (1.2)	0.002	5.7 (1.2)	0.009	5.8 (1.2)	< 0.001

Data are reported as mean (SD), unless otherwise stated

Results were not included for 116 individuals who were clinically detected screening participants but had missing information on OGTT results (glycaemic status) from previous screening

Unconfirmed screen-positive individuals only had a diabetic screening result, whereas confirmed screen-detected individuals had a diabetic screening result and a medical or prescription record of diabetes within 1 year. Those with clinically detected diabetes were identified in five sources of medical and prescription records, unrelated to screening

Missing data: BMI, *n* = 49; self-reported health, *n* = 78; systolic BP, *n* = 82; diastolic BP, *n* = 83; serum total cholesterol, *n* = 61

*p* values are for comparisons with screen-detected diabetes

The standardised mortality rate was 3.2/1000 person-years for all VIP participants, 8.4/1000 person-years for all non-participants and 3.0/1000 person-years for known normoglycaemic VIP participants (data not shown). Average follow-up time was 8.7 (median 7.8) years for individuals with screen-detected diabetes, and 7.2 (median 6.2) years for those who were clinically detected diabetes, after their date of detection (maximum 21.9 years). Those with screen-detected diabetes had a consistently lower rate of all-cause mortality, CVD, renal disease and retinopathy than those with clinically detected diabetes (Table 3). Among clinically detected individuals with diabetes, screening participants had lower rates of all outcomes compared with screening non-participants. There was a clear pattern of HR; compared with screen-detected diabetes, those who had clinically detected diabetes who were screening participants had an increased risk of poor health outcomes (e.g. all-cause mortality HR 1.70 [95% CI 1.32, 2.18]), and clinically detected individuals who were screening non-participants had an even higher risk of poor health outcomes (e.g. all-cause mortality HR 2.31 [95% CI 1.82, 2.94]) (Table 4). The results were similar over the course of follow-up; a total of 3397 (30.7%) individuals with diabetes, including 394 screen-detected individuals, were followed for 10 years or longer. The HR for all-cause mortality after 10 years was 1.91 (95% CI 1.27, 2.85) for clinically detected vs screen-detected diabetes cases.

**Table 3:** Crude and standardised incident event and mortality rates among individuals with type 2 diabetes mellitus, VIP 1992–2013

Variable	Deaths			CVD events			Renal disease			Retinopathy		
	*n*	MR	StdMR	*n*	IR	StdIR	*n*	IR	StdIR	*n*	IR	StdIR
Confirmed screen-detected diabetes	73	8.2	4.2	128	15.5	8.7	39	4.4	3.3	70	8.1	6.3
Unconfirmed screen-positive individuals	139	10.4	5.9	141	11.0	5.8	23	1.7	0.9	9	0.7	0.4
Incident clinically detected diabetes	1330	21.4	15.5	1704	30.5	21.9	649	10.8	9.3	757	12.7	12.7
Screening participants	515	18.8	11.5	680	27.1	19.8	258	9.6	8.6	279	10.5	9.7
Previous diabetes-indicative OGTT > 1 year before detection	93	17.0	9.4	114	22.9	13.7	41	7.7	5.0	70	13.3	9.9
Previous non-diabetic hyperglycaemia	194	16.4	12.0	267	24.5	21.0	100	8.7	10.5	103	9.0	10.3
Previous normoglycaemia	206	22.0	12.5	275	32.3	20.4	102	11.2	7.9	91	10.0	8.8
Screening non-participants	815	23.4	18.4	1024	33.3	25.5	391	11.6	10.2	478	14.6	14.2

Incidence rate and mortality rate are reported per 1000 person-years. Age- and sex-standardised mortality rate and incidence rate are calculated with the total study population as reference

Rates do not include events that coincide with the date of diabetes detection

CVD, renal and retinal events were detected in the National Patient Register and the Cause of Death Register

Unconfirmed screen-positive individuals only had a diabetic screening result, whereas confirmed screen-detected individuals had a diabetic screening result and a medical or prescription record of diabetes within 1 year. Those with clinically detected diabetes were identified in five sources of medical and prescription records, unrelated to screening

IR, incidence rate; MR, mortality rate; StdIR, standardised incidence rate; StdMR, standardised mortality rate

**Table 4:** Associations between mode of detection of type 2 diabetes mellitus and death, incident CVD events, renal disease or retinopathy, VIP 1992–2013

Variable	All-cause mortality, HR (95% CI)	CVD events, HR (95% CI)	Renal disease, HR (95% CI)	Retinopathy, HR (95% CI)
Confirmed screen-detected diabetes	1 (Ref)	1 (Ref)	1 (Ref)	1 (Ref)
Unconfirmed screen-positive individuals	1.35 (1.01, 1.79)	0.77 (0.60, 0.98)	0.41 (0.25, 0.69)	0.08 (0.04, 0.16)
Incident clinically detected diabetes	2.07 (1.63, 2.62)	1.55 (1.29, 1.86)	2.26 (1.64, 3.13)	1.66 (1.30, 2.13)
Screening participants	1.70 (1.32, 2.18)	1.25 (1.03, 1.52)	1.89 (1.34, 2.66)	1.38 (1.06, 1.80)
Previous diabetes-indicative OGTT > 1 year before detection	1.61 (1.18, 2.20)	1.11 (0.86, 1.43)	1.58 (1.02, 2.45)	1.73 (1.24, 2.42)
Previous non-diabetic hyperglycaemia	1.59 (1.21, 2.09)	1.18 (0.95, 1.46)	1.76 (1.21, 2.56)	1.19 (0.87, 1.61)
Previous normoglycaemia	2.05 (1.56, 2.70)	1.47 (1.19, 1.83)	2.20 (1.51, 3.20)	1.35 (0.98, 1.85)
Screening non-participants	2.31 (1.82, 2.94)	1.77 (1.47, 2.13)	2.54 (1.82, 3.53)	1.85 (1.44, 2.38)

HRs adjusted for calendar time in time scale and the covariates age at diabetes detection, calendar year of diabetes detection, sex and SES reported in the 1990 census

SESs imputed for *n* = 1561 individuals who had missing information

Unconfirmed screen-positive individuals had only a diabetic screening result, whereas confirmed screen-detected individuals had a diabetic screening result and a medical or prescription record of diabetes within 1 year. Those with clinically detected diabetes were identified in five sources of medical and prescription records, unrelated to screening

Ref, reference population

Among clinically detected individuals with diabetes who were screening participants, those who had had previous normoglycaemia tended to have higher rates of adverse health outcomes, with the exception of retinopathy, compared with those who had had previous OGTTs indicative of diabetes or non-diabetic hyperglycaemia (Table 3). In general, those who had had previous OGTT measurements in the diabetic range and those who had had previous non-diabetic hyperglycaemia had very similar HRs (e.g. all-cause mortality HR 1.61 and 1.59, respectively), whereas those who had had previous normoglycaemia had higher risks for all outcomes (e.g. all-cause mortality HR 2.05 [95% CI 1.56, 2.70]), with the exception of retinopathy (Table 4).

To explore whether some of the effect of mode of detection of diabetes among participants in screening could be explained by differences in an individual’s health status, we conducted sensitivity analyses adjusting for presence of a prior CVD event, and additionally for several biomarkers measured at previous screening (listed in Table 2), as well as time since previous screening (ESM Table 2). As a result, when adjusting for prior CVD event status, all estimates were attenuated but, with the exception of the HR of CVD events (HR 1.16 [95% CI 0.95, 1.41]), remained significant. Estimates were further attenuated when adjusting for additional biomarkers.

Unconfirmed screen-positive individuals were on average 1.7 years younger at the date of their diabetes-indicative OGTT screening result than those who had a confirmed diagnosis (Table 1); they had a higher mortality rate than for screen-detected diabetes, but a lower incidence rate for all other outcomes, and a very low incidence rate for renal disease and retinopathy (Table 3). Compared with confirmed screen-detected diabetes cases, unconfirmed screen-positive cases had higher risk of all-cause mortality (HR 1.35 [95% CI 1.01, 1.79]) but lower risk of CVD (HR 0.77 [95% CI 0.60, 0.98]) and substantially lower HR of renal disease (HR 0.41 [95% CI 0.25, 0.69]) and retinopathy (HR 0.08 [95% CI 0.04, 0.16]) (Table 4).

## Discussion

In this study of a population included in an organised universal screening programme for diabetes, we found that a diagnosis of diabetes can be brought forward by an average of 4.6 years by screening asymptomatic individuals, and that screen-detected individuals appear to fare better than those with clinically detected diabetes after their diagnosis.

The lead time is somewhat longer than the 3.3 years and 2.2 years estimated in previous studies [5, 7]. There are important differences with regards to screening interval and analytical approach between this study and the previous studies that may explain this difference. In the Ely cohort, one-third of the population was randomly invited to participate in screening for diabetes in 5 year intervals (screened population), and two-thirds of the cohort were not. However, at the third screening round, one-third of the population initially not included in the screening arm were randomly invited to take part (‘unscreened’ population). Lead time was calculated as the difference in median diabetes duration for the screened and ‘unscreened’ population, both of which included screen- and clinically detected individuals. In the ADDITION-Denmark study, high-risk individuals were invited to screening at one time-point, and lead time was calculated as the difference in median diabetes duration between screen-detected individuals vs. clinically detected individuals in the whole group eligible to be screened. In this study, we compared age at detection in individuals with screen-detected vs clinically detected diabetes who had been eligible to be screened in 10 year intervals.

We found that those with screen-detected diabetes had lower rates of all-cause mortality and incident CVD, renal disease and retinopathy than those with clinically detected diabetes. This is in line with the modelled estimated reduction in CVD events caused by earlier routine treatment that was found in a previous study [24]. It is possible that the observed effect may be caused by the treatment that screen-detected individuals presumably received earlier than those whose diabetes had been clinically detected, but there are three important biases that may explain some of the effect.

The first is healthy user bias. Clinically detected individuals who were screening non-participants were detected on average 3.6 years earlier than clinically detected individuals who were screening participants, but despite being diagnosed with diabetes earlier, they had consistently worse health outcomes. On average, VIP non-participants had more than twice the rate of all-cause mortality than VIP participants when comparing age- and sex-standardised mortality rates. Similarly, it has been shown that in screening for human papilloma virus, regular non-attenders have about a twofold higher all-cause mortality than regular attenders [25]. Although VIP is not a screening programme for diabetes, it is likely that the individual choice to attend the clinical examinations would be guided by similar behaviour to the choice to attend systematic organised screening programmes. In the VIP, participation has been linked to marital status and higher income, but not education [12].

Second, there is length time bias. The idea that slowly developing disease with a longer asymptomatic preclinical screen-detectable course is also more likely to have a long clinical course and better prognosis [26] has not previously been explored specifically for diabetes. However, our data indicate that this concept may be equally important for diabetes screening as it is for several cancers [27]. It appears that slowly progressing hyperglycaemia and diabetes may be associated with better health outcomes than more rapidly progressing disease, as indicated by the fact that clinically detected individuals with diabetes who had normoglycaemia at their previous VIP examination had worse health outcomes after diagnosis than those who had been non-diabetic hyperglycaemic or who had had diabetes-indicative OGTT results. However, those who had diabetic or non-diabetic hyperglycaemia at their previous screening should have received lifestyle advice and referrals to continued care, which could have also contributed to a better prognosis after diagnosis. In addition, the mean time to diabetes detection from screening was about 4 years longer for those with previous normoglycaemia, and we cannot know for how long they would have lived with hyperglycaemia prior to their diagnosis. Ideally, we would have liked to test the contribution of length time bias by adjusting for health status and biomarkers for diabetes severity at the time of detection, but these data were not available. When we adjusted for several biomarkers associated with general health status measured at the previous screening, the estimates were attenuated, which supports a role of length time bias, but the analysis has limitations so a cautious interpretation is warranted.

The third source of bias is lead time bias. Although we adjusted for age at detection in the analyses, we cannot disregard the fact that there may be residual bias from differences in lead time as screen-detected individuals had on average a 1.5 years longer observation time owing to being detected earlier in the disease course than those with clinically detected diabetes [28, 29].

Taken together, these data suggest that there may be a positive effect of early detection and treatment due to screening on survival and health outcomes after a diagnosis of diabetes, but how much is not within the scope of this study to determine. These results are in line with those from ADDITION-Denmark [7].

We found that it was more common for a diabetes-indicative OGTT result to remain unconfirmed than to be confirmed within 1 year, which is in line with findings from previous studies [8]. In this study, unconfirmed screen-positive individuals were overall younger and had consistently better health at the point of the positive diabetic screening result than confirmed screen-detected individuals with diabetes. They also had lower incidence rates of CVD and renal disease, a considerably lower incidence rate of retinopathy, but a higher mortality rate. There is reason to believe that some of the difference in retinopathy and renal disease rates is due to surveillance bias as individuals with confirmed diabetes are more likely to be tested for these conditions, but this is less likely to be the case for CVD events and is not the case for deaths. These results indicate that unconfirmed screen-positive individuals would potentially benefit from treatment for the management of blood glucose levels and related risk factors in order to reduce the risk of CVD [30].

The primary strengths of this study were the large population size and the fact that we were able to study a model for an organised whole population-based screening programme for diabetes with follow-up for over 20 years that included participants as young as age 30 years. The main limitations were the relatively short follow-up period and that we could not assess the association between screening in general and health outcomes after diagnosis owing to the non-randomised design (lack of a non-screening control group). The diagnostic criteria for diabetes were revised during the study period when the fasting glucose level threshold in the OGTT was lowered from 7.8 mmol/l to 7.0 mmol/l in 1999 [31], although it was unclear when this revision was implemented in the VIP, meaning that some individuals may have been misclassified. However, there were only ten screen-detected individuals (data not shown) within this range between 1992 and 1998, and since all diabetes cases in this group were confirmed by another source within 1 year, the resulting bias is likely to be limited. The median follow-up time was relatively short at 6.2–7.8 years, but the results were similar even after 10 years’ follow-up. We did not have access to data on marital status and income, variables that have been associated with propensity to participate in screening [12], but we were able to control for SES. Individuals with clinically detected diabetes were identified from five different sources, and as a consequence systematic information on biomarkers associated with severity of diabetes at time of diagnosis was unavailable.

In conclusion, in this population-based study of screen- and clinically detected diabetes, we found that screen-detected individuals were detected at a younger age, and may have better survival and lower rates of CVD, renal disease and retinopathy than those who were clinically detected.

## Electronic supplementary material

ESM Tables (PDF 115 kb)

## Abbreviations

ADDITION: Anglo–Danish–Dutch study of intensive treatment in people with screen-detected diabetes in primary care
CVD: Cardiovascular disease
SES: Socioeconomic status
VIP: Västerbotten Intervention Programme
