# Supplementary material for: Screening for type 2 diabetes: do screen-detected cases fare better?
Source: Diabetologia. 2017 Aug 23;60(11):2200–9. doi: 10.1007/s00125-017-4402-4 (PMC6086324; doi:10.1007/s00125-017-4402-4)
Supplement: Supplementary file 1 — (PDF 115 kb) [file 125_2017_4402_MOESM1_ESM.pdf]

Electronic Supplementary Material (ESM) to
"Screening for type 2 diabetes: do screen-detected cases fare better?"
By Feldman AL, *et al.*

ESM Table 1. International Classification of Diseases (ICD) codes used to identify health outcome events in the Swedish Cause of Death and National Patient Register (including inpatient and outpatient hospital records).

Type of event		ICD-9	ICD-10	Description
Cardiovascular disease (CVD)	Myocardial infarction	410	I21	Acute myocardial infarction
			I22	Subsequent myocardial infarction
			I23	Certain current complications following acute myocardial infarction
		411	I24	Other acute (and subacute) forms of ischemic heart disease
	Heart failure	428	I50	Heart failure
		402A/01	I11.0	Hypertensive heart disease with (congestive) heart failure
	Stroke	430	I60	Subarachnoid haemorrhage
		431	I61	Intracerebral haemorrhage
		432	I62	Other and unspecified intracranial haemorrhage
			I63	Cerebral infarction
			I64	Stroke, not specified as haemorrhage or infarction
		433	I65	Occlusion (and stenosis) of precerebral arteries
		434	I66	Occlusion (and stenosis) of cerebral arteries
	Peripheral arterial disease	250G/7	E11.5<br>E13.5<br>E14.5	Diabetes with peripheral circulatory disorders
		440C/2	I70.2	Atherosclerosis of arteries of extremities
		443X/9	I73.9	Peripheral vascular disease, unspecified
		443W/8	I79.2	Peripheral angiopathy in diseases classified elsewhere
Renal disease		403A/01	I12.0	Hypertensive renal disease with renal failure
		404	I13	Hypertensive heart and renal disease
		584	N17	Acute renal failure
		585	N18	Chronic kidney disease (CKD)
		586	N19	Renal failure, unspecified
		250D/4	E11.2<br>E13.2<br>E14.2	Diabetes with renal manifestations
Retinopathy		362D/3	H34	Retinal vascular occlusions
		362B/1<br>362G/6<br>362W/8<br>362X/9	H35.0<br>H35.4<br>H35.6<br>H35.9	Background retinopathy and retinal vascular changes<br>Peripheral retinal degeneration<br>Retinal haemorrhage<br>Retinal disorder, unspecified
		362A/0	H36.0	Diabetic retinopathy
		250E/5	E11.3<br>E13.3<br>E14.3	Diabetes with ophthalmic manifestations

Electronic Supplementary Material (ESM) to
"Screening for type 2 diabetes: do screen-detected cases fare better?"
By Feldman AL, *et al.*

ESM table 2. Sensitivity analyses among screening participants only: Associations between mode of detection of diabetes and death, incident CVD events, renal complications, or retinal complications. Västerbotten Intervention Programme 1992-2013.

	All-cause mortality		CVD events		Renal Disease		Retinopathy	
	HR	95% CI	HR	95% CI	HR	95% CI	HR	95% CI
Main analysis adjusted for prior CVD event status	1.65	1.28, 2.12	1.16	0.95, 1.41	1.83	1.30, 2.58	1.39	1.07, 1.82
Main analysis adjusted for prior CVD event status, time between previous screening and detection, biomarkers measured at previous screening	1.42	1.05, 1.92	1.01	0.79, 1.28	1.51	1.00, 2.29	1.07	0.77, 1.49

HRs are for incident clinically detected individuals with diabetes who were screening participants vs. screen-detected individuals (reference).

HRs adjusted for calendar time in time-scale and the co-variates age at diabetes detection, calendar year of diabetes detection, sex, and socioeconomic status reported in the 1990 census in addition to variables specified above. Biomarkers at previous screening were BMI, systolic and diastolic blood pressure, total serum cholesterol and self-reported overall bad health.

Socioeconomic status imputed for n=1,565 individuals who had missing information.

CI: Confidence interval, CVD: Cardiovascular disease, HR: Hazard ratio, OGTT: Oral glucose tolerance test.
